# Assembly and comparative analysis of the first complete mitochondrial genome of *Acer truncatum* Bunge: a woody oil-tree species producing nervonic acid

**DOI:** 10.1186/s12870-021-03416-5

**Published:** 2022-01-13

**Authors:** Qiuyue Ma, Yuxiao Wang, Shushun Li, Jing Wen, Lu Zhu, Kunyuan Yan, Yiming Du, Jie Ren, Shuxian Li, Zhu Chen, Changwei Bi, Qianzhong Li

**Affiliations:** 1 Institute of Leisure Agriculture, Jiangsu Academy of Agricultural Sciences, Nanjing, 210014 China; 2 Nanjing Forestry University, Nanjing, 210037 China; 3 Institute of Agricultural Engineering, Anhui Academy of Agricultural Sciences, 40 Nongkenanlu, Hefei, 230031 Anhui China

**Keywords:** *Acer truncatum*, Mitochondrial genome, Repeats, Phylogenetic analysis

## Abstract

**Background:**

*Acer truncatum* (purpleblow maple) is a woody tree species that produces seeds with high levels of valuable fatty acids (especially nervonic acid). The species is admired as a landscape plant with high developmental prospects and scientific research value. The *A. truncatum* chloroplast genome has recently been reported; however, the mitochondrial genome (mitogenome) is still unexplored.

**Results:**

We characterized the *A. truncatum* mitogenome, which was assembled using reads from PacBio and Illumina sequencing platforms, performed a comparative analysis against different species of *Acer*. The circular mitogenome of *A. truncatum* has a length of 791,052 bp, with a base composition of 27.11% A, 27.21% T, 22.79% G, and 22.89% C. The *A. truncatum* mitogenome contains 62 genes, including 35 protein-coding genes, 23 tRNA genes and 4 rRNA genes. We also examined codon usage, sequence repeats, RNA editing and selective pressure in the *A. truncatum* mitogenome. To determine the evolutionary and taxonomic status of *A. truncatum*, we conducted a phylogenetic analysis based on the mitogenomes of *A. truncatum* and 25 other taxa. In addition, the gene migration from chloroplast and nuclear genomes to the mitogenome were analyzed. Finally, we developed a novel *NAD1* intron indel marker for distinguishing several *Acer* species.

**Conclusions:**

In this study, we assembled and annotated the mitogenome of *A. truncatum*, a woody oil-tree species producing nervonic acid. The results of our analyses provide comprehensive information on the *A. truncatum* mitogenome, which would facilitate evolutionary research and molecular barcoding in *Acer*.

**Supplementary Information:**

The online version contains supplementary material available at 10.1186/s12870-021-03416-5.

## Background

*Acer truncatum* Bunge (Sapindaceae) is a versatile, oil-producing woody tree widely distributed mainly in northern China, Japan and Korea [1, 2]. This tree species is a potential source of medicinal compounds, including flavonoids, alkaloids, tannins, and terpenoids [3]. Moreover, *A. truncatum* seed oil contains approximately 90% unsaturated fatty acids and was listed as a new food resource by the Ministry of Health of the People’s Republic of China in 2011 [2]. Nervonic acid (24:15*, cis*-15-tetracosenoic acid, n-9) accounts for 5–6% of seed oil [2, 4]. It is a key component of brain nerve cells as well as tissues promoting the repair and regeneration of nerve cells and damaged tissues. Previous studies have indicated that nervonic acid is potentially useful for treatment of schizophrenia, psychosis, and attention deficit disorder [5, 6]. It has been detected in several plant species [2, 7, 8], but issues related to their nervonic acid content and growth adaptability have limited the utility of these species. The characteristics of rapid growth, wide geographic distribution, and high adaptability, thus *A. truncatum* is a novel potential plant source of nervonic acid for treating human cerebral and neurological problems.

The main function of mitochondria, the “energy factories” of cells, is the conversion of biomass energy into chemical energy in living cells [9, 10]. In most seed plants, nuclear hereditary information is inherited biparentally, whereas DNA of both mitochondria and chloroplasts is maternally derived [9, 10]. In addition, recent researches have revealed that intergenomic gene transfer between nuclear and organellar genomes, which was a common phenomenon during plant evolution [11–13]. Along with rapid developments in sequencing and genome assembly technologies, an increasing amount of information on mitogenomes has been uncovered. At present, 6026 complete land plant organelle genomes, including 5735 chloroplast and 291 plant mitogenomes have been assembled and deposited in GenBank Organelle Genome Resources (https://www.ncbi.nlm.nih.gov/genome/browse/), as the mitochondrial genome is more complex and harder to assemble than that of other organelles [9, 14].

Plant mitogenomes are species specific [15, 16] and vary considerably in length, gene order, and gene content [9, 10, 14, 17]. Genome size is extremely variable, ranging from 66 kb (*Viscum scurruloideum*) [18] to 11.3 Mb (*Silene conica*) [19], and most genomes are 200–800 kb in size [20]. This wide variation in mitogenome size can be attributed to the repetitive sequences and the foreign DNA from other organisms during evolution [21, 22]. Repetitive sequences, including simple sequence repeats (SSRs), tandem repeats and dispersed repeats, are abundant in the mitogenomes of seed plants. SSRs are frequently used as molecular markers for identifying species in plant mitogenomes [14, 23]. In addition, insertions/deletions (indels) and single nucleotide polymorphisms (SNPs) within mitogenomes also have been applied to rapidly distinguish species and for phylogenetic analyses [24, 25].

The mitochondrial gene content of land plants varies considerably, ranging from 32 to 67 genes. Some genes, including those related to NADH dehydrogenase, ATP synthase, ubiquinol cytochrome, and cytochrome c biogenesis [14], are highly conserved, whereas others, such as *sdh3*, *sdh4*, *rps11*, and *cox2* have been lost [26, 27].

Mitogenomes in the genus *Acer*, except for the mitogenome sequence of *A. yangbiense* released in 2019, have not been analyzed in detail [28]. In this study, we first assembled the complete mitogenome of *A. truncatum* and analyzed its gene content, repetitive sequences, RNA editing sites, selective pressure, and phylogenetic relationships. We also surveyed gene transfer among nuclear, chloroplast, and mitochondrial genomes of *A. truncatum*. Moreover, we developed a marker based on an indel in the *NAD1* intron to distinguish seven *Acer* species (*A. buergerianum*, *A. truncatum*, *A. henryi*, *A. negundo*, *A. ginnala*, *A. yangbiense* and *A. tonkinense*). The data presented herein expand genetic information available for the genus *Acer* and provide an opportunity to conduct further important genomic breeding studies on *A. truncatum*.

## Results

### Features of the *A. truncatum* mitogenome

**Fig. 1:**
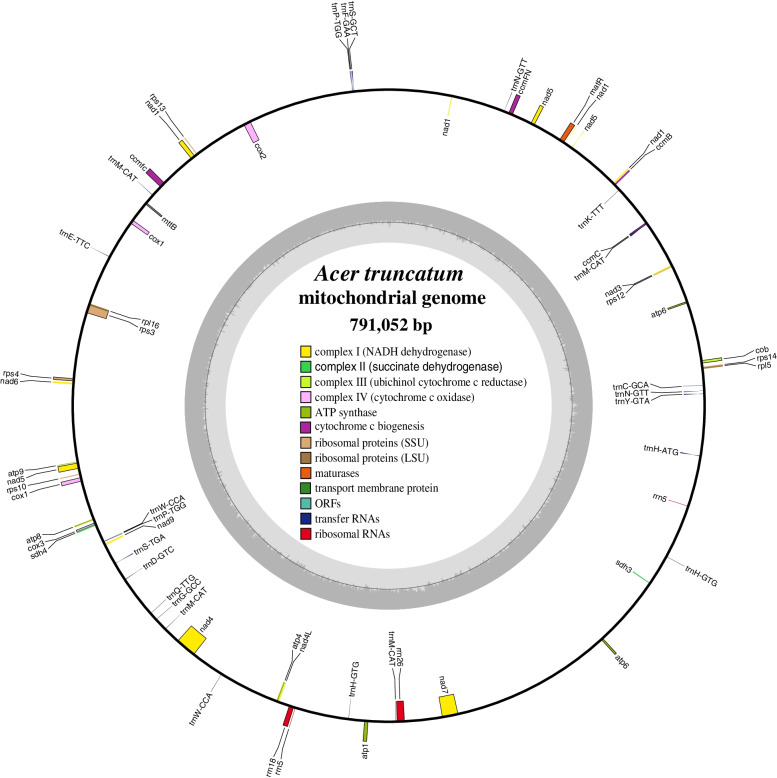
Circular map of the *A. truncatum* mitogenome. Genes shown on the outside and inside of the circle are transcribed clockwise and counterclockwise, respectively. The dark gray region in the inner circle depicts GC content. Asterisks besides genes denote intron-containing genes

The *A. truncatum* genome sequence generated was submitted to the GenBank database (accession number MZ318049) in this study. The complete mitogenome of *A. truncatum* is 791,052 bp in length and has the typical circular structure of land plant genomes (Fig. 1). The nucleotide composition of the complete mitogenome is 27.11% A, 27.21% T, 22.79% G, and 22.89% C, with a GC content of 45.68% (Table 1). Protein-coding genes (PCGs) and *cis* introns account for 4.31 and 2.94% of the whole mitogenome, while tRNA and rRNA genes comprise only 0.22 and 0.67%, respectively. A total of 62 unique genes, including 35 protein-coding, 23 tRNA, and 4 rRNA genes, were identified in the *A. truncatum* mitogenome (Table 2). Interestingly, two copies of *cox1* genes were found. Additionally, five tRNA and one rRNA gene(s) located in repeat sequences were found to be present in two or four copies (*trnN-GTT*, *trnM-CAT*, *trnP-TGG*, *trnH-GTG*, *trnW-CCA*, and *rrn5*) (Fig. 1).

**Table 1:** Genomic features of the *A. truncatum* mitogenome

Feature	A(%)	C(%)	G(%)	T(%)	GC(%)	Size (bp)	Proportion in Genome (%)
Whole genome	27.11	22.89	22.79	27.21	45.68	791, 052	100
Protein-coding genes	26.12	31.1	21.52	21.25	52.62	34, 059	4.31
cis-spliced introns	23.59	26.56	26.56	24.76	53.11	23, 222	2.94
tRNA genes	24.59	26.27	24.77	24.36	51.04	1728	0.22
rRNA genes	23.37	26.34	25.74	24.55	52.08	5280	0.67
Non-coding regions	27.30	22.36	22.36	27.60	44.71	72, 6763	91.87

**Table 2:** Gene profile and organization of the *A. truncatum* mitogenome

Group of genes	Gene name	Length	Start codon	Stop codon	Amino acids
ATP synthase	*atp1*	1530	ATG	TGA	509
	*atp4*	597	ATG	TAG	198
	*atp6*	774	ACG	TAA	257
	*atp8*	480	ATG	TAA	159
	*atp9*	225	ATG	TGA	74
NADH dehydrogenase	*nad1*^*a*^	978	ACG	TAA	325
	*nad2*^*a*^	1467	ATG	TAA	488
	*nad3*	357	ATG	TAA	118
	*nad4*^*a*^	1488	ATG	TGA	495
	*nad4L*	303	ACT	TAA	100
	*nad5*	2004	ATG	TAA	667
	*nad6*	618	ATG	TAA	205
	*nad7*^*a*^	1185	ATG	TAG	394
	*nad9*	573	ATG	TAA	190
Cytochrome c biogenesis	*ccmB*	621	ATG	TGA	206
	*ccmC*	753	ATG	TGA	250
	*ccmFc*^*a*^	1365	ATG	TAG	454
	*ccmFn*	1734	ATG	TGA	577
Maturases	*matR*	1962	ATG	TAG	653
Ubichinol cytochrome c reductase	*cob*	1182	ATG	TGA	393
Cytochrome c oxidase	*cox1(2)*	1584	ATG	TAA	527
	*cox2*	795	ATG	TGA	264
	*cox3*	798	ATG	TGA	265
Transport membrane protein	*mttB*	792	ATA	TAG	264
Ribosomal proteins (LSU)	*rpl5*	555	ATG	TAA	184
	*rpl16*	516	ATG	TAA	171
Ribosomal proteins (SSU)	*rps3*^*a*^	1686	ATG	TAA	561
	*rps4*	1077	ATG	TAA	358
	*rps10*^*a*^	330	ATG	TAA	109
	*rps12*	378	ATG	TGA	125
	*rps13*	294	ATG	TGA	97
	*rps14*	255	ATG	TGA	84
Succinate dehydrogenase	*sdh3*	327	ATG	TGA	108
	*sdh4*	480	ATG	TAA	159
Transfer RNAs	*trnY-GTA*	83	_	_	_
	*trnN-GTT*^*b*^*(2)*	72	_	_	_
	*trnC-GCA*	71	_	_	_
	*trnM-CAT(4)*	73/74/74/77	_	_	_
	*trnK-TTT*	73	_	_	_
	*trnS-GCT*	88	_	_	_
	*trnF-GAA*	74	_	_	_
	*trnP-TGG*^*b*^*(2)*	74/75	_	_	_
	*trnE-TTC*	72	_	_	_
	*trnW-CCA*^*b*^*(2)*	73/74	_	_	_
	*trnS-TGA*	87	_	_	_
	*trnD-GTC*^*b*^	74	_	_	_
	*trnQ-TTG*	72	_	_	_
	*trnG-GCC*	72	_	_	_
	*trnH-GTG*^*b*^*(2)*	74/74	_	_	_
	*trnH-ATG*	76	_	_	_
Ribosomal RNAs	*rrn5(2)*	119/120	_	_	_
	*rrn18*	1939	_	_	_
	*rrn26*	3102	_	_	_

Note: Numbers after gene names are the number of copies. The superscripts a and b indicate genes containing introns and chloroplast-derived genes, respectively

### Codon usage analysis of PCGs

**Fig. 2:**
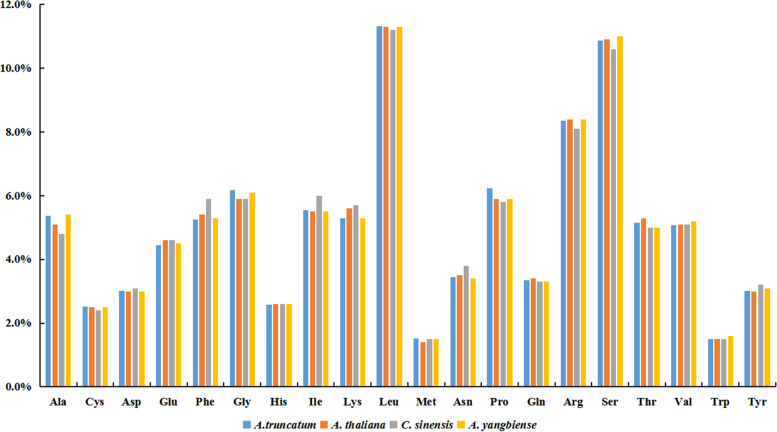
Codon usage pattern of the *A. truncatum* mitogenome compared with *A. yangbiense*, *A. thaliana*, and *C. sinensis*. The relative percentage of each amino acid residue in all mitochondrial proteins is shown on the *y*-axis

The total length of PCGs in *A. truncatum* was 34,059 bp. Most PCGs had the typical ATG start codon, whereas *atp6*, *nad1*, and *nad4L* had ACG as the start codon—presumably a consequence of C-to-U RNA editing of the second site (Table 2). Three types of stop codons were identified, namely, TAA, TGA, and TAG, the C to U RNA editing phenomenon was not found in the stop codons. As shown in Fig. 2, the codon usage analysis revealed the most frequent amino acids to be leucine (Leu) (11.2–11.3%), serine (Ser) (10.6–11.0%), and arginase (Arg) (8.1–8.4%), whereas cysteine (Cys) and tryptophan (Trp) were rarely found.

**Fig. 3:**
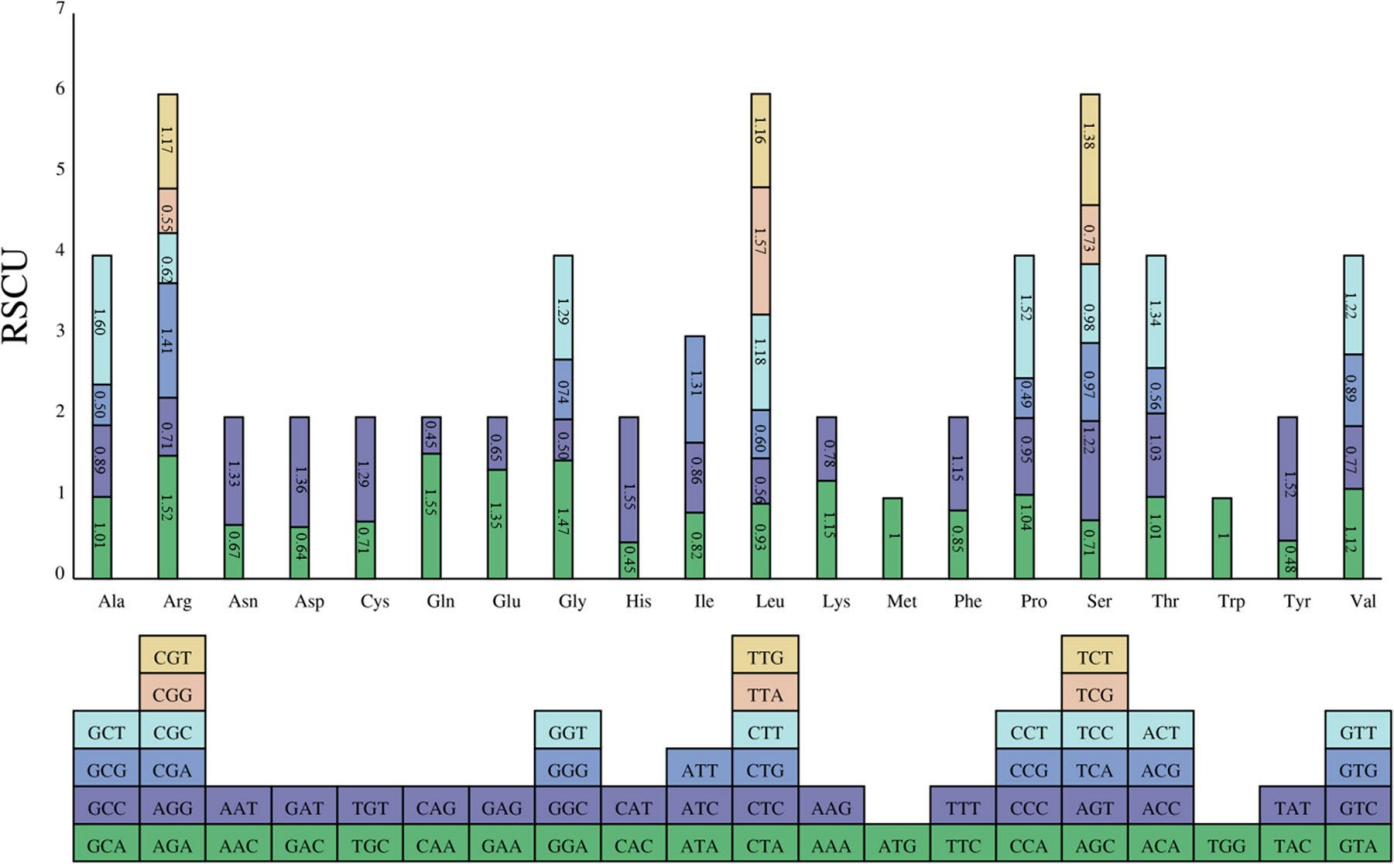
Relative synonymous codon usage (RSCU) in the *A. truncatum* mitogenome. Codon families are shown on the *x*-axis. RSCU values are the number of times a particular codon is observed relative to the number of times that codon would be expected for a uniform synonymous codon usage

We also analyzed the relative synonymous codon usage (RSCU) of 35 PCGs in the *A. truncatum* mitogenome. As shown in Fig. 3, the 35 PCGs comprised 33,948 bp encoding 11,316 codons excluding termination codons. We found that nearly all of the RSCU values of NNT and NNA codons were higher than 1.0 with the exception of Ile (AUA, 0.82), Leu (CUA, 0.93), and Ser (UCA, 0.97). Codon usage was generally strongly biased toward A or T(U) at the third codon position in the *A. truncatum* mitogenome, which is very common in mitogenomes of land plant species.

### Analysis of synonymous and nonsynonymous substitution rates

**Fig. 4:**
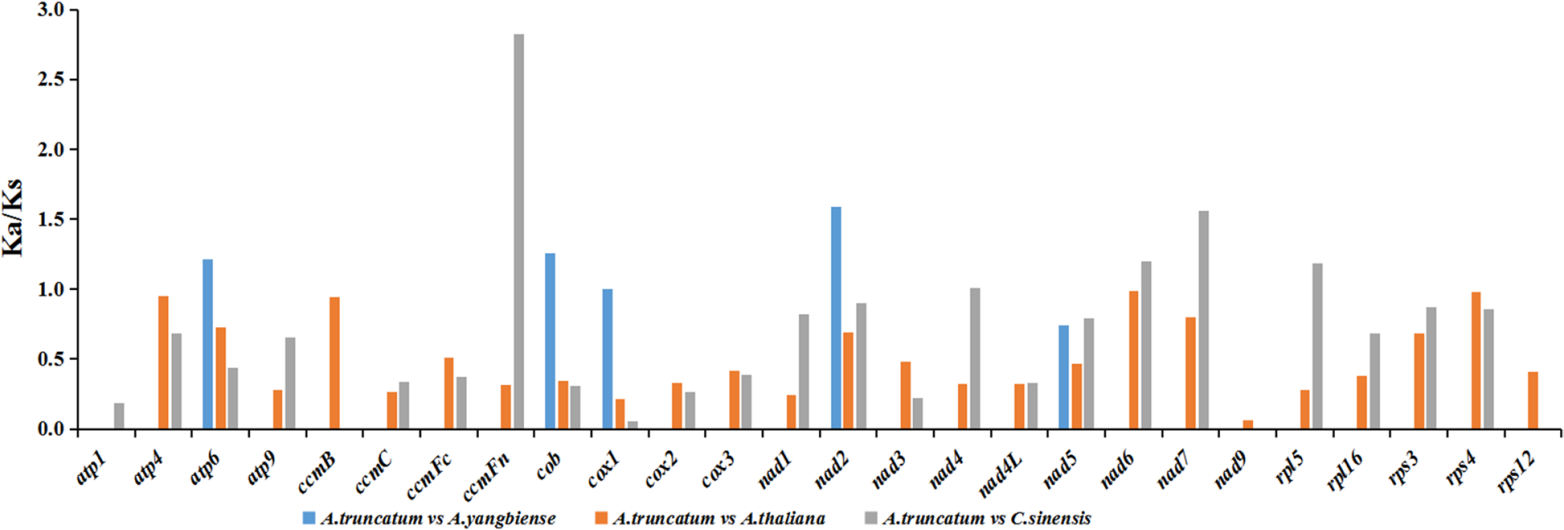
Ka/Ks ratios of 26 protein-coding genes in *A. truncatum*, *A. yangbiense*, *A. thaliana*, and *C. sinensis*

In genetics, the nonsynonymous-to-synonymous substitution ratio (*Ka/Ks*) is used to understand the evolutionary dynamics of genes. In this study, the *Ka/Ks* ratio was determined for 26 protein-coding genes common to *A. truncatum*, *A. yangbiense*, *A. thaliana* and *C. sinensis* mitogenomes (Fig. 4). The PCGs shared between *A. truncatum* and *A. yangbiense* were close homologs, as the *Ka/Ks* ratio of 21 PCGs was 0. In addition, nearly all *Ka/Ks* ratios were less than 1.0, which suggested that most of the PCGs were subject to stabilizing selection during evolution. Conversely, the *Ka/Ks* ratios of nine genes (*atp6*, *cob*, *cox1*, *nad2*, *ccmFn*, *nad4*, *nad6*, *nad7* and *rpl5*) were greater than 1.0, which indicateed these genes had been under positive selection during evolution. Finally, three genes (*atp4*, *ccmB* and *rps4*) had *Ka/Ks* ratios close to 1, thus suggested that they had experienced neutral evolution since the divergence of their common ancestor.

### Prediction of RNA editing sites in PCGs

**Fig. 5:**
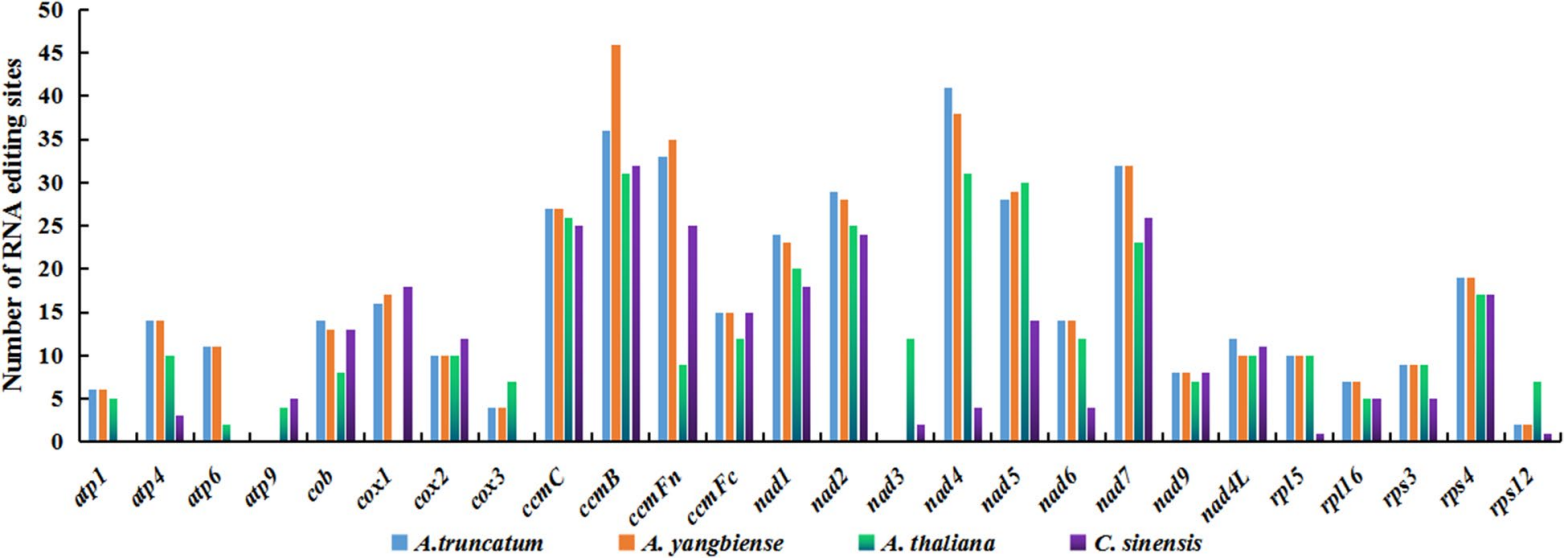
The distribution of RNA editing sites in mitogenome protein-coding genes of four angiosperms

In plants, RNA editing is necessary for gene expression, with cytidine (C)-to-uridine (U) RNA editing enriched in mitochondrial and chloroplast genomes. In this study, we predicted the RNA editing sites of 26 PCGs common to mitogenomes of four angiosperm species. The number of RNA editing sites predicted for *A. truncatum*, *A. yangbiense*, *A. thaliana*, and *C. sinensis*—421, 427, 342 and 288, respectively—suggests that these sites are extremely conserved in PCGs in *Acer*. A total of 421 RNA editing sites were predicted in *A. truncatum*, all exhibiting C-to-U RNA editing. Among the 421 sites, 32.07 and 67.93% were predicted at the first and the second positions of codons, respectively, whereas none were found at the third position (Fig. 5).

RNA editing can change PCG initiation and termination codons. As shown in Table 2, *atp6*, *nad1* and *nad4L* genes use ACG as their initiation codons, we infer that they may have been altered by RNA editing. The number of RNA editing sites in different genes was found to vary greatly, with the largest predicted numbers detected in cytochrome *c* biogenesis (*ccmB*, *ccmC*, *ccmFn*, and *ccmFc*), *Complex I* (NADH dehydrogenase) and *nad4* genes. In contrast, no RNA editing sites were found in *atp9* and *nad3* genes in *A. truncatum* and *A. yangbiense*.

### Analysis of repeats in the *A. truncatum* mitogenome

**Fig. 6:**
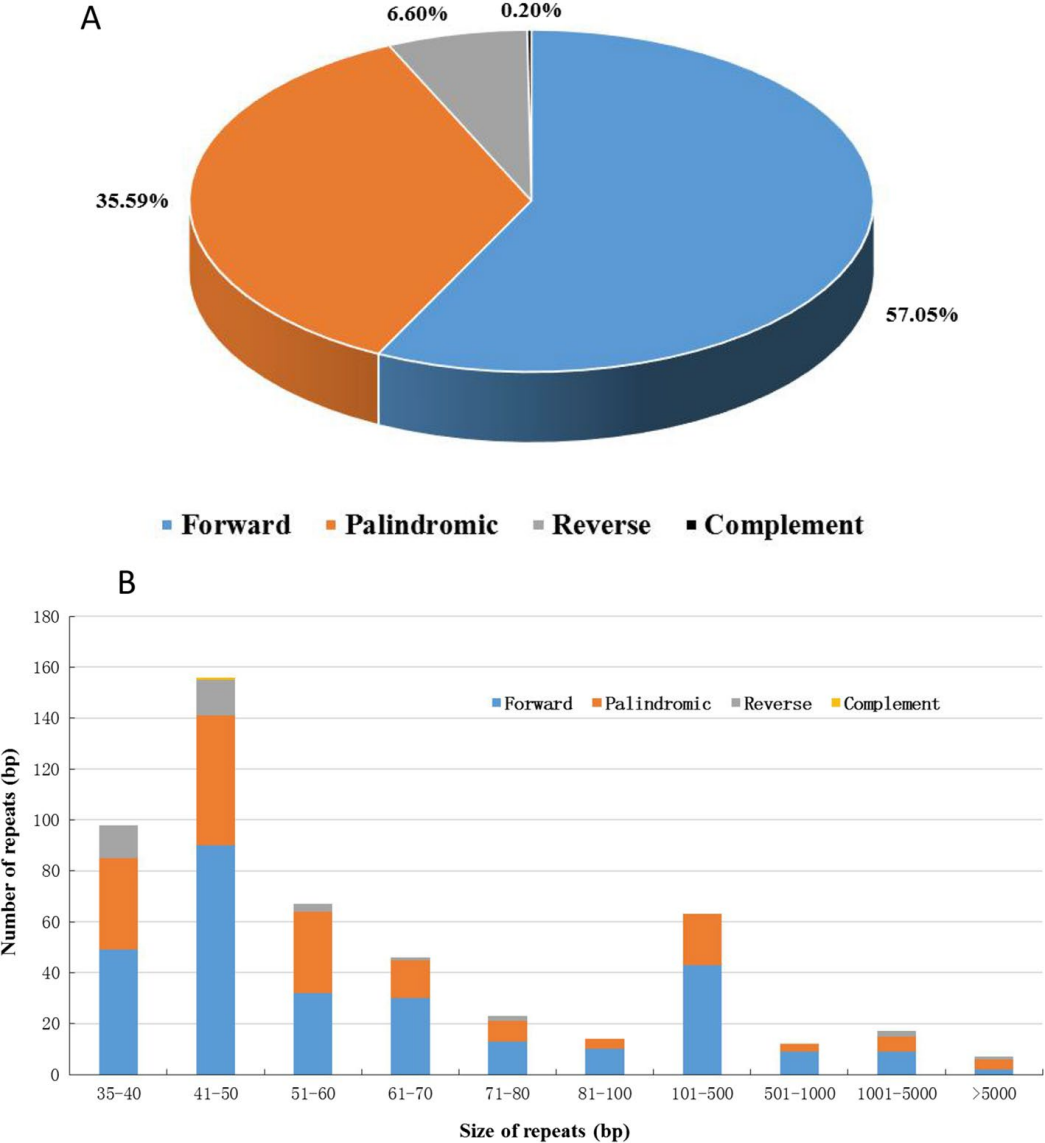
Detected repeats in the *A. truncatum* mitogenome. **A** Type and proportion of detected repeats. **B** Frequency distribution of repeat lengths

An analysis of repeats in the *A. truncatum* mitogenome revealed 503 long repeats (> 30 bp), namely, 287 forward (57.05%), 179 palindromic (35.59%), 33 reverse (6.60%) and 1 complementary (0.20%) repeats (Fig. 6A). The total length of the long repeats was 144,318 bp, which corresponded to 18.24% of the mitogenome. Most repeats were 35–50 bp long (254 repeats, 50.29%), whereas 24 were longer than 1 kb, the largest was 28,452 bp (Fig. 6B and Table S1). In *A. truncatum* mitogenome, we found that five pair of large repeats (> 1 kb) by rearrangements could produce two subgenomic circles, which comprising of 457,840 bp and 333,212 bp, mediated by the pairwise large repeats R3a and R3b (Table S2 and Fig.S1). We also identified repeats in the *A. yangbiense* mitogenome to further characterize repeats in *Acer* species. A total of 500 long repeats were found, including 271 forward (54.20%), 88 palindromic (17.60%), and 141 reverse (28.20%) repeats. No complementary repeats were identified. The total length constituted by long repeats was 138,024 bp, which accounted for 17.18% of the *A. yangbiense* mitogenome (803, 281 bp) (Fig. S2A and B). Most repeats were 41–60 bp long (288 repeats, 57.60%), the longest repeat was 27,124 bp (Table S3).

SSRs, which are tandem repeated sequences with motifs of one to six bases, are useful molecular markers for studying genetic diversity and identifying species [14, 23]. In this study, a total of 717 SSRs were detected in the *A. truncatum* mitogenome, including 226 (31.52%) mono-, 355 (49.51%) di-, 49 (6.83%) tri-, 67 (9.34%) tetra-, 18 (2.51%) penta-, and 2 (0.28%) hexanucleotide repeats (Table 3). Among the 717 SSRs, more than 81% were mono- and di-repeats. Further analysis of SSR repeat units indicated that 85.40% of monomers had A/T contents, and 45.07% of dinucleotide repeats were AT/TA. The higher AT content of SSRs contributed to the AT richness (54.32%) of the complete *A. truncatum* mitogenome.

**Table 3:** Frequency of identified SSR motifs in the *A. truncatum* mitogenome

Motif Type	Number of repeats													Total	Proportion (%)
	3	4	5	6	7	8	9	10	11	12	14	15	21		
**Monomer**	–	–	–	–	–	118	65	28	7	3	2	2	1	226	31.52
**Dimer**	–	280	51	14	5	2	2	1	–	–	–	–	–	355	49.51
**Trimer**	–	43	3	1	1		1	–	–	–	–	–	–	49	6.83
**Tetramer**	59	7	1	–	–	–	–	–	–	–	–	–	–	67	9.34
**Pentamer**	16	–	–	2	–	–	–	–	–	–	–	–	–	18	2.51
**Hexamer**	2	–	–	–	–	–	–	–	–	–	–	–	–	2	0.28
**Total**	77	330	55	17	6	120	68	29	7	3	2	2	1	717	100

### Phylogenetic analysis

**Fig. 7:**
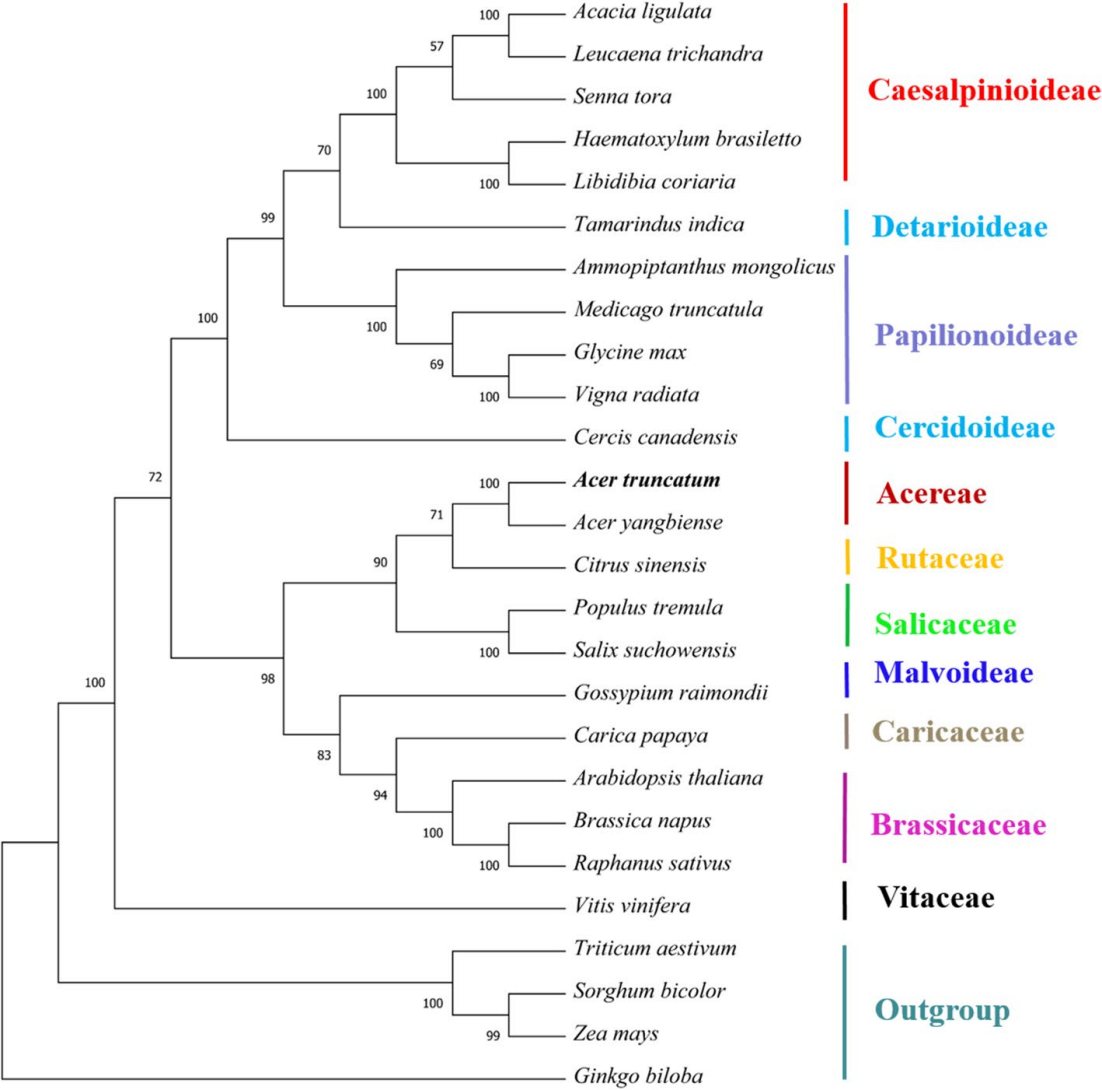
Maximum-likelihood phylogenetic tree based on 25 single-copy orthologous genes shared among 26 species. Numbers at nodes are bootstrap support values. The position of *A. truncatum* is indicated in bold. *Triticum aestivum*, *Sorghum bicolor, Ginkgo biloba*, and *Zea mays* served as outgroups

To determine the phylogenetic position of *A. truncatum*, we downloaded 25 plant mitogenomes from GenBank (https://www.ncbi.nlm.nih.gov/genome/browse/) (Table S4) and constructed a phylogenetic tree based on a set of 25 conserved single-copy orthologous genes present in all 26 analyzed mitogenomes. As shown in Fig. 7, 21 of 23 nodes in the generated tree had bootstrap support values over 70%, including 12 nodes with 100% support. The phylogenetic tree strongly supports (100% bootstrap support) the close phylogenetic relationship between *A. truncatum* and *A. yangbiense*. In addition, it also revealed that both species were closely related to *C. sinensis*, which is similar to conclusions inferred using the nuclear genome [2]. Overall, the results of our analysis of mitogenomes provide a valuable foundation for future analyses of the phylogenetic affinities of *Acer* species.

### Plastid-derived and nuclear-shared sequence transfer events

**Fig. 8:**
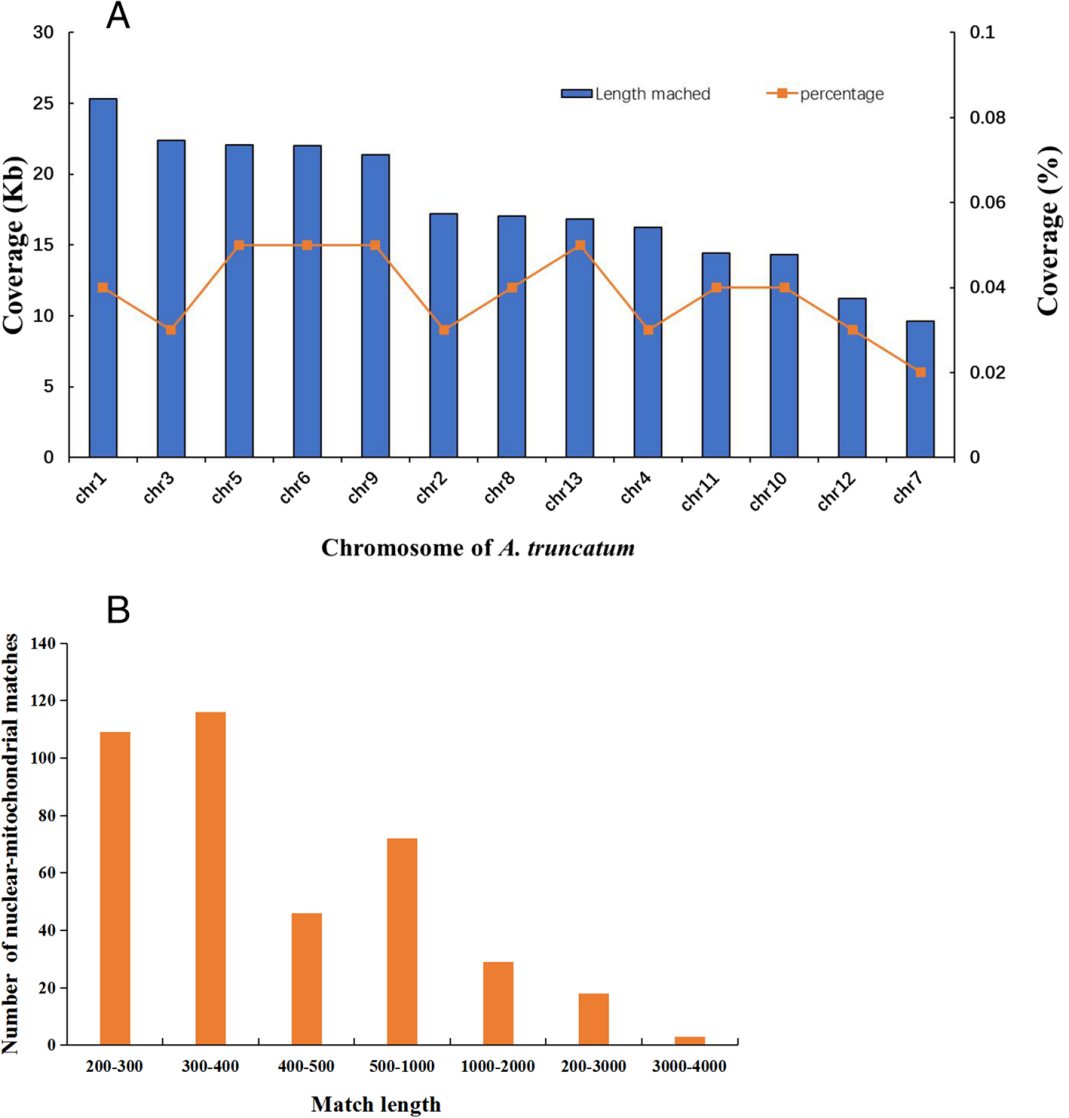
Characteristics of nuclear–mitochondrial sequences in *A. truncatum*. **A** Distributions of percent identities between shared nuclear–mitochondrial matches. The number of matches is shown by blue boxes and is plotted on the left ordinate. The orange lines, which represent the coverage of matches on nuclear and mitochondrial genomes, are plotted on the right ordinate. **B** Distributions of lengths between shared nuclear–mitochondrial matches

DNA fragment transfers among nuclear and organellar genomes are common events during plant evolution. Six directions of gene transfer are possible among the three types of genomes. To further understand the characteristics of sequence transfer events in *A. truncatum*, the *A. truncatum* nuclear and chloroplast genomes [2, 29] were searched by using its mitogenome sequences as queries. We obtained 393 hits covering 230.0 kb of sequences of nuclear genome transferred into the mitogenome. According to the nuclear–mitochondrial alignment, hits occurred on every *A. truncatum* chromosome (Fig. 8A), however, the total lengths of the hits and the percent coverage on the chromosomes were different. Chromosome 1 had the maximum total length of hits (25.30 kb), which was much larger than on other chromosomes, whereas the highest percent coverage (0.05%) occurred on chromosomes 5, 6, and 13. In addition, fragment lengths were mainly between 200 bp and 400 bp (Fig. 8B). A total of 62,241 bp of sequences (7.87% of the *A. truncatum* mitogenome) were found to be shared between nuclear and mitochondrial genomes. The shared sequences contained seven complete genes (*trnN-GTT*, *rpl5*, *trnS-GCT*, *trnF-GAA*, *trnQ-TTG*, *atp1*, and *trnH-GTG*) as well as partial gene sequences of *matR*, *ccmFN*, *cox2*, *rps3*, *rps4*, *atp8*, *sdh4*, *nad4* and *atp6*.

The *A. truncatum* mitogenome sequence (791,052 bp) was approximately five times longer than the chloroplast genome (156,492 bp). Forty-one fragments with a total length of 18,637 bp, corresponding to 2.36% of the mitogenome, were observed to have migrated from the chloroplast genome to the mitogenome in *A. truncatum* (Table 4). Six intact chloroplast genes (*psbJ*, *trnP-UGG*, *trnW-CCA*, *trnN-GUU*, *trnD-GUC*, and *trnH-GUG*) were located on these fragments*.* The remaining fragments were partial sequences of transferred genes or intergenic spacer regions in the chloroplast genome. Interestingly, we found that the DNA migration had often occurred in the inverted repeat region of the *A. truncatum* chloroplast genome.

**Table 4:** Fragments transferred from chloroplasts to mitochondria in *A. truncatum*

	Alignment Length	Identity %	Mismatch	Gap opens	CP Start	CP End	Mt Start	Mt End	Gene
1	2890	99.689	9	0	22,796	25,685	588,995	586,106	*rpoC1*
2	2890	99.239	21	1	22,796	25,685	762,940	760,052	*rpoC1*
3	2700	99.963	1	0	20,067	22,766	699,997	697,298	
4	1259	97.935	7	8	99,134	100,376	576,186	574,931	
5	1259	97.935	7	8	142,127	143,369	574,931	576,186	
6	1259	97.935	6	9	142,127	143,369	730,678	729,424	
7	1259	97.935	6	9	99,134	100,376	729,424	730,678	
8	1067	90.909	55	28	65,889	66,926	449,842	450,895	*psbJ*
9	351	99.715	1	0	45,259	45,609	437,233	437,583	*ycf3*
10	349	99.713	1	0	75,224	75,572	46,646	46,994	*psbB*
11	224	99.107	1	1	138,184	138,407	589,548	589,326	*trnI-GAU*
12	224	99.107	1	1	104,096	104,319	589,326	589,548	*trnI-GAU*
13	205	93.171	12	2	66,231	66,434	362,782	362,579	*psbF*
14	173	94.798	9	0	68,227	68,399	451,672	451,844	*trnP-UGG*
15	141	100	0	0	110,171	110,311	150,810	150,670	
16	141	100	0	0	132,192	132,332	150,670	150,810	
17	131	100	0	0	35,802	35,932	277,631	277,761	*psbC*
18	123	92.683	9	0	67,987	68,109	451,461	451,583	*trnW-CCA*
19	93	100	0	0	136,588	136,680	694,605	694,513	*trnA-UGC*
20	93	100	0	0	105,823	105,915	694,513	694,605	*trnA-UGC*
21	98	97.959	2	0	110,389	110,486	150,666	150,569	
22	98	97.959	2	0	132,017	132,114	150,569	150,666	*trnN-GUU*
23	178	84.831	17	8	30,780	30,955	468,861	469,030	*trnD-GUC*
24	105	96.19	3	1	109,900	110,003	533,208	533,104	
25	105	96.19	3	1	132,500	132,603	533,104	533,208	
26	90	98.889	1	0	151,306	151,395	452,466	452,377	*ycf2*
27	90	98.889	1	0	91,108	91,197	452,377	452,466	*ycf2*
28	83	100	0	0	59,800	59,882	72,900	72,982	*accD*
29	86	96.512	3	0	132,009	132,094	5982	5897	*trnN-GUU*
30	86	96.512	3	0	110,409	110,494	5897	5982	*trnN-GUU*
31	79	98.734	1	0	54,466	54,544	494,502	494,424	*trnM-CAU*
32	75	100	0	0	154,389	154,463	77,164	77,090	*trnI-CAU*
33	75	100	0	0	88,040	88,114	77,090	77,164	*trnI-CAU*
34	80	97.5	2	0	7	86	577,140	577,219	*trnH-GUG*
35	80	97.5	2	0	7	86	728,470	728,391	*trnH-GUG*
36	72	100	0	0	101,734	101,805	206,176	206,105	
37	72	100	0	0	140,698	140,769	206,105	206,176	
38	77	93.506	4	1	7420	7495	131,293	131,217	
39	56	100	0	0	87,421	87,476	346,975	346,920	*rpl2*
40	56	100	0	0	155,027	155,082	346,920	346,975	*rps12(exon)*
41	65	92.308	2	3	54,754	54,816	448,383	448,446	*atpE*
Total	18,637								

### Development of an *NAD1* intron indel marker

**Fig. 9:**
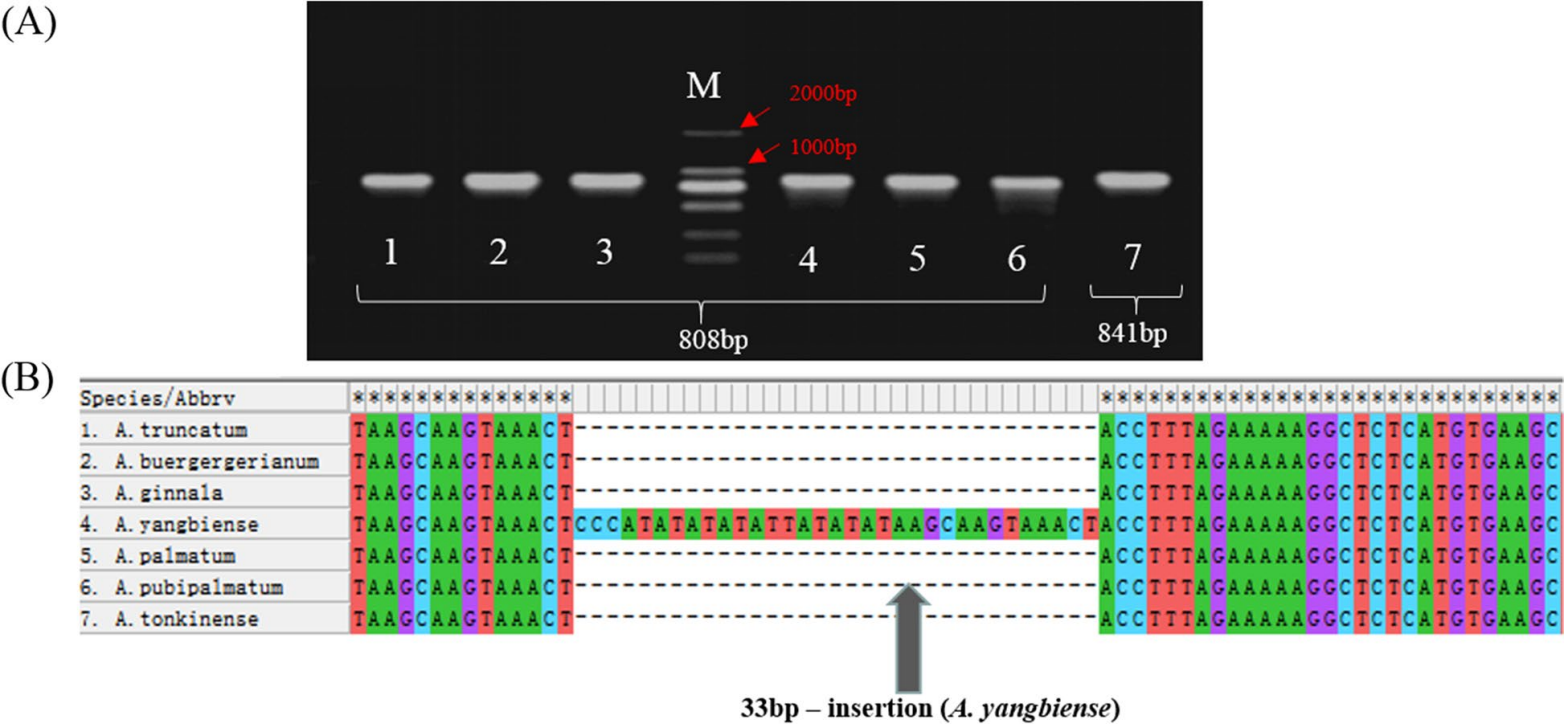
Schematic diagram of the development of an *NAD1* intron marker in seven *Acer* species. **A** Electrophoretic gel visualization of the amplified fragments. Lanes are as follows: 1, *A. tonkinense*; 2, *A. ginnala*; 3, *A. pubipalmatum*; 4, *A. palmatum*; 5, *A. truncatum*; 6, *A. buergerianum*; 7*, A. yangbiense*; M, 2000-bp ladder. **B** Alignment of the *NAD1* intron marker sequence in MEGA 6.0. The arrow indicates the 33-bp insertion in *A. yangbiense*

Among *Acer* species, only the mitogenome of *A. yangbiense* has currently been reported. To further characterize the *NAD1* intron*,* we compared its sequence between *A. truncatum* and *A. yangbiense*, and detected a 33-bp indel. The following seven *Acer* species were selected for characterization of the *NAD1* intron sequence: *A. truncatum*, *A. buergerianum*, *A. ginnala*, *A. yangbiense*, *A. palmatum*, *A. pubipalmatum*, and *A. tonkinense*. To develop indel markers, primers were designed to anneal to conserved regions of the *NAD1* intron (Table S5). The predicted amplification products were successfully obtained using these *NAD1*-intron-F/R primers in all seven tested samples (Fig. 9A). In all six species, the length of the amplified *NAD1* intron sequence was identical (808 bp) and highly conserved. The corresponding sequence in *A. yangbiense* was indeed longer (841 bp) because of the 33-bp putative insertion (Fig. 9A and B). Several species close to *A. truncatum* in the phylogenetic tree (*A. yangbiense*, *Populus tremula*, *Salix suchowensis* and *C. sinensis*) were selected to verify whether the 33-bp sequence was an insertion or a deletion. According to the sequence alignment, the sequence was indeed an insertion (Fig. S3). In previous studies, indel markers have frequently been used to distinguish closely related species; however, *Acer* species have not been identified on the basis of their mitogenomes using this approach. Our first-ever characterization of the *NAD1* intron in *Acer* may therefore be applicable for classification and identification of *Acer* species.

## Discussion

### Characterization of the *A. truncatum* mitochondrial genome

Mitochondria, which produce the energy required to carry out life processes, are the powerhouses of plants. Because of factors such as size variation and repeated sequences, plant mitogenomes are more complex than those of animals [9, 14, 30, 31]. The emergence of rapid, cost-effective genome sequencing technologies has accelerated understanding of mitogenomes. Our study has produced the first detailed characterization of a complete mitogenome in *Acer*. The size of the *A. truncatum* mitogenome is similar to that of *A. yangbiense* [28], both of which are moderate in size relative to most genomes [32]. GC content is an important factor for assessing species. The GC content of the *A. truncatum* mitogenome is 45.68%, which is comparable to that of other sequenced plant mitogenomes (*A. thaliana*, 44.8% [33]; *Phaseolus vulgaris*, 45.11% [14]; *Beta vulgaris*, 43.9% [34], but higher than the *A. truncatum* chloroplast genome (37.90%) assembled by our research group [29]. Similar to most other mitogenomes, most sequences in the *A. truncatum* mitogenome are non-coding. Protein-coding genes account for only 4.31%, which is probably the result of a gradual increase in sequence duplication during evolution. Most PCGs were the typical ATG start codon, and the distribution of amino acid compositions was similar to other angiosperms [28, 33]. while the *atp6*, *nad1* and *nad4L* genes use ACG as initiation codons, this phenomenon also has been found in other studies, which were considered to be altered by RNA editing modification [9, 14]. The *cox1* is one of the most reported mitochondrial genes involved in horizontal gene transfer among sngiosperms. In our study, two copies of *cox1* genes were found. The previous studies reported that the *cox1* copies existed in different species and different populations of a species [35]. Ka/Ks ratios > 1 have also been reported for some other mitochondrial genes [9, 14, 26]. In our study, the high Ka/Ks ratios of genes observed were very important for further studies in the gene selection and evolution of *Acer* species, including *atp6*, *cob*, *cox1*, *nad2*, *ccmFn* etc.

### Identification of repeat sequences and RNA editing sites

Repeats are important sources of information for developing markers for population and evolutionary analyses [23, 36, 37]. Including tandem, short and large repeats, they are widely present in mitogenomes [14, 38, 39]. Repeats in mitochondrial DNA are generally vital for intermolecular recombination, which can generate structural variations and extreme mitogenome sizes [20, 40]. In this study, five pair of large repeats (> 1 kb) by rearrangements could produce two subgenomic circles in *A. truncatum* mitogenome, comprising of 457,840 bp and 333,212 bp, respectively. This phenomenon also reported in Soybean [41]. we also found major differences between the repeat sequences of *A. truncatum* and *A. yangbiense* mitogenomes. In particular, the proportion of long repeat sequences in the *A. truncatum* mitogenome (18.24%) was higher than that of *A. yangbiense* (17.20%), and the longest repeats were 28,452 bp and 27,124 bp, respectively. These repeats may have contributed to the increase in the mitogenome size of *A. yangbiense.* This finding also suggests that intermolecular recombination has frequently occurred in the mitogenome during *Acer* evolution [14, 31].

RNA editing, a post-transcriptional process that occurs in chloroplast and mitochondrial genomes of higher plants, contributes to improved protein folding [9, 14, 26]. Previous researches had uncovered approximately 491 RNA editing sites within 34 genes in rice [42] and 486 RNA editing sites within 31 genes in *P. vulgaris* [14]. In the present study, we predicted RNA editing sites in 26 PCGs common to *A. truncatum*, *A. yangbiense*, *A. thaliana* and *C. sinensis* mitogenomes. We found that the number of RNA editing sites in PCGs was extremely conserved in *Acer* but differed in the other two species. Although the number of RNA editing sites varies greatly among genes, cytochrome *c* biogenesis and NADH dehydrogenase genes harbor the largest number, which is similar to *P. vulgaris* [14]. In addition, all identified RNA editing sites are located at first and second codon positions. Previous researchers have speculated that the lack of RNA editing sites at the third codon position is probably due to the limitations of the PREP-Mt predictive methodology used rather than an actual absence [14, 43]. Further analysis using experimental methods is thus needed.

### DNA fragment transfer events

Information pertaining to DNA transfer events between different genomes (mitochondrial, nuclear and chloroplast) has been uncovered by sequencing analysis [21, 44, 45]. Previous studies have determined that the most prominent transfer direction in angiosperms is from organellar genomes into the nuclear genome, followed in importance by transfer from nuclear and plastid genomes into the mitogenome [13, 21, 46–48]. The total length of transferred DNA varies among plant species in higher plants, lengths range from 50 kb (*A. thaliana*) to 1.1 Mb (*O. sativa* subsp. *japonica*) [49]. According to our study, 230.0 kb of nuclear DNA has been transferred into the mitogenome of *A. truncatum*. Although the nuclear–mitochondrial transfer process has occurred on every *A. truncatum* chromosome, the total lengths of transferred material and the percent coverage differs among chromosomes. In total, 62, 241 bp of sequences (7.87% of the *A. truncatum* mitogenome) is shared between nuclear and mitochondrial genomes. Most genes with transferred sequences shared between nuclear and mitochondrial genomes are tRNA genes, such as *trnN-GTT, trnH-GTG*, and *trnH-GTG*. Chang et al. [41] have reported similar results in soybean. In regards to chloroplast genome to mitogenome migration events, a total of 18,637 bp of transferred fragments were observed, accounting for 2.36% of the *A. truncatum* mitogenome. In comparison, the proportion in *S. suchowensis* and *Suaeda glauca* is 2.8 and 5.18%, respectively [31]. We identified 41 fragments that had been transferred from the chloroplast genome to the mitogenome, these fragments included six integrated genes, namely, five tRNA genes and *psbJ.* Transfer of tRNA genes from chloroplast to mitochondrial DNA is common in angiosperms [21, 26, 31]. Interestingly, we also observed that DNA migration often occurred in the inverted repeat region of the *A. truncatum* chloroplast genome.

### Development of a mitochondrial *NAD1* intron marker for *Acer* species

Because indel regions are relatively easy to detect, they are often used to develop markers for identifying species [50]. The genus *Acer* comprises more than 200 species grown in China [2, 51]; however, the highly similar shapes of some species present a challenge for identification, and a molecular approach would be beneficial. *NAD1* intron indel markers have been useful for identification of some plant species [52–54]. In *Acer*, only the mitogenome of *A. yangbiense* has been previously reported [28]. In the present study, we first identified a 33-bp sequence difference by aligning the *NAD1* intron regions of *A. truncatum* and *A. yangbiense*. Amplification of the *NAD1* intron with specific primers revealed that a 33-bp indel was present in *A. yangbiense*, whereas the amplified *NAD1* intron sequence was of the same length and highly conserved in the other six species. We verified that this 33-bp indel was an insertion in *Acer* by analyzing several species close to *A. truncatum* in our phylogenetic tree (*A. yangbiense*, *P. tremula*, *S. suchowensis*, and *C. sinensis*). The development of mitogenome-based molecular markers has not been previously reported for *Acer*. Although only a few *Acer* species were used in this study, our findings should nonetheless contribute to species classification in *Acer*.

## Conclusions

In this study, we assembled and annotated the mitogenome of *A. truncatum* and performed extensive analyses based on DNA and amino acid sequences of annotated genes. The *A. truncatum* mitogenome is circular, with a length of 791,052 bp. We annotated 62 genes, including 35 protein-coding, 23 tRNA and 4 rRNA genes. In addition, the codon usage, sequence repeats, RNA editing and selective pressure were also analyzed in the *A. truncatum* mitogenome. The evolutionary status of *A. truncatum* was verified by phylogenetic analysis based on the mitogenomes of this species and 25 other taxa. Gene conservation between chloroplast and mitochondrial genomes and between nuclear and mitochondrial genomes were also detected in *A. truncatum* by analyzing gene migration. Finally, a newly developed *NAD1* intron indel marker was used to distinguish *Acer* species. Our study has yielded extensive information about the *A. truncatum* mitogenome. The data presented herein supplement the genetic knowledge available for the genus *Acer*, provide novel insights into *A. truncatum* evolution, and form an important theoretical basis for increasing *A. truncatum* seed yield.

## Materials and methods

### Plant materials and DNA sequencing

*A. truncatum* plants were grown at our Aceraceae seed base of Jangsu Academy of Agricultural Sciences (Lishui District, Nanjing, China; 31°65 N, 119°02E) under natural conditions. Fresh leaves were frozen in liquid nitrogen and stored at 80 °C. DNA extraction and sequencing were performed using methods described in our previous de novo genome sequencing study [2].

### Mitogenome assembly and annotation

For the *A. truncatum* mitogenome, PacBio RS II reads (59.42 GB) sequenced in our previous study [2] were de novo assembled using Canu v1.4 [55]. The obtained contigs were mapped to core mitochondrial genes by minimap2 [56], then extended. The assembled contigs were polished (Pilon v1.18) with Illumina reads (75.0 GB) to correct read errors [57]. Finally, five large contigs were assembled into mitogenome, to verify the quality and accuracy of our assemblies, we further verified the junctions by Sanger sequencing (Table S6 and Fig. S4). The GE-Seq tool on the MPI-MP CHLOROBOX website (https://chlorobox.mpimp-golm.mpg.de) was used for the mitogenome annotation, with the *A. yangbiense* mitogenome (CM017774.1) serving as a reference. Mitochondrial protein-coding genes were predicted using the MITOFY webserver [40]. All tRNA genes were confirmed by using tRNAscan-SE with default settings [9, 14, 58]. ORFfinder (https://www.ncbi.nlm.nih.gov/orffinder/) was used to analyze open reading frames longer than 300 bp. RSCU values and the amino acid composition of PCGs were calculated in MEGA X [59]. A circular mitochondrial map was drawn using Organellar Genome DRAW [60].

### Analysis of repeat structures and SSRs

Forward, reverse, palindromic and complementary repeats were identified with REPuter [61]. SSRs were analyzed with the MISA program [62]. The motif size of one- to six- nucleotide SSRs was set as 8, 4, 4, 3 and 3, respectively.

### Selective pressure analysis

We calculated the nonsynonymous (Ka) and synonymous (Ks) substitution rates of each PCG between *A. truncatum* and *A. yangbiense*, *A. thaliana*, and *C. sinensis*. Orthologous gene pairs were separately aligned in MEGA 6.0. Ka, Ks, and Ka/Ks values were calculated using DnaSP [63].

### Genome alignments

The *A. truncatum* mitogenome was searched against the chloroplast genome of *A. truncatum* (MH638284) using BLASTN 2.9.0+ according to the following screening criteria: matching rate ≥ 70%, E-value ≤1e^− 6^, and length ≥ 40 [31]. To identify regions of potential nuclear origin in the mitogenome of *A. truncatum*, we also performed a BLASTN search (maximum E-value = 1e^− 50^) of the complete mitogenome against all contigs from the *A. truncatum* nuclear genome sequenced in our previous study. BLASTN results of sequences longer than 250 bp and a pairwise similarity *>* 80% were inspected for sequence features.

### Prediction of RNA editing sites

RNA editing sites in the PCGs of *A. truncatum* and other three mitogenomes (*A. yangbiense*, *A. thaliana* and *C. sinensis*) were predicted using the the online PREP-Mt suite of servers (http://prep.unl.edu/). To obtain a more accurate prediction, the cutoff value was set as 0.2 [43]..

### Phylogenetic analyses

A total of 26 complete mitogenomes (Table S4) were used to ascertain the phylogenetic position of *A. truncatum*. The 25 mitochondrial PCG genes (*atp1*, *atp4*, *atp6*, *atp8*, *atp9*, *ccmB, ccmC*, *ccmFc*, *ccmFn*, *cob*, *cox1*, *cox3*, *matR*, *nad1*, *nad2*, *nad3*, *nad4*, *nad4L*, *nad5*, *nad6*, *nad7*, *nad9*, *rps12*, *rps3*, and *rps4*) conserved across the 26 analyzed species were aligned in Muscle with default parameters [64], with the alignment then modified manually to eliminate gaps and missing data. Finally, a maximum likelihood tree was constructed in MEGA X using the JTT + G + I + F nucleotide substitution model [58]. A bootstrap consensus tree was inferred from 1000 bootstrap replicates. *Triticum aestivum*, *Sorghum bicolor, Ginkgo biloba*, and *Zea mays* were used as outgroups.

### Verification of the *Nad1* insertion in *Acer*

Primers were designed with Primer 5. PCR amplifications were carried out in 15-μl volumes containing 20 ng genomic DNA, 0.4 μl dNTPs (2.5 mM each), 2.5 μl of 10× Ex *Taq* buffer (Mg^2+^), 0.4 μl Ex *Taq* DNA polymerase (Takara,Tokyo, Japan), and 1.0 μl of each primer (10 mM). The amplification conditions were 94 °C for 5 min, followed by 30 cycles of 94 °C for 30 s, 56 °C for 30 s, and 72 °C for 30 s, with a final extension of 72 °C for 10 min. The PCR products were purified and linked to the pMD19-T easy plasmid (Takara) for sequencing to confirm the accuracy of PCR product sizes. Three samples per species were sequenced by the General Biology Company (Nanjing, Jiangsu, China).

## Supplementary Information

**Additional file 1: Figure S1.** Reversible reorganization of the *A. truncatum* mitgenome may produce subgenomic circles by large repeats. The same colour triangles represent the pairs of large repeats.

**Additional file 2: Figure S2.** The type of detected repeats and the frequency distribution of lengths in the *A. yangbiense.*

**Additional file 3: Figure S3.** Alignment of the *NAD1* intron sequence with MEGA-X.

**Additional file 4: Figure S4.** Agarose gel electrophoresis of PCR product for contig connecting verification

**Additional file 5: Table S1.** The repeat sequences distributions in the *A. truncatum* mitogenome genome.

**Additional file 6: Table S2.** The large repeats (> 1 kb) by rearrangements could produce two subgenomic circles in *A. truncatum* mitogenome.

**Additional file 7: Table S3.** The repeat sequences distributions in the *A. yangbiense* mitogenome genome.

**Additional file 8: Table S4.** Details regarding the mitochondira genome sequences used for the phylogenetic analysis.

**Additional file 9: Table S5.** Details regarding the primers used to develop the *NAD1* intron marker.

**Additional file 10: Table S6.** Primers for contig connecting verification

## Data Availability

The *A. truncatum* Mitochondrial genome sequence was deposited in the GenBank database (accession number MZ318049).

## Abbreviations

Ile: Isoleucine
Leu: Leucine
Ser: Serine
PCR: Polymerase chain reaction
SSRs: Simple sequence repeat
PCG: Protein-coding genes
DNA: Deoxyribonucleic acid
SNPs: Single Nucleotide Polymorphism
tRNA: Tranfer RNA
rRNA: Ribosomal RNA
Arg: Arginine
Cys: Cysteine
Trp: Tryptophan
RSCU: Relative synonymous codon usage
*Ka/K*: *S*nonsynonymous-to-synonymous substitution ratio.

## References

[1] Guo X, Wang R, Chang R, Liang X, Wang C, Luo Y (2014). Effects of nitrogen addition on growth and photosynthetic characteristics of *Acer truncatum* seedlings. Dendrobiology..

[2] Ma Q, Sun T, Li S, Wen J, Zhu L, Yin T (2020). The *Acer truncatum* genome provides insights into nervonic acid biosynthesis. Plant J.

[3] Tang W, Wang J, Xu J, Wang L, Huang J, Chen Y (2012). Advances of chemical composition of medicinal plants in Aceraceae. Northern Horticulture.

[4] Wang X, Wang S (2005). A new resource of nervonic acid from purpleblow maple (*Acer truncatum*) seed oil. For Prod J.

[5] Tanaka K, Shimizu T, Ohtsuka Y, Yamashiro Y, Oshida K (2007). Early dietary treatments with Lorenzo’s oil and docosahexaenoic acid for neurological development in a case with Zellweger syndrome. Brain and Development.

[6] Amminger G, Schäfer M, Klier C, Slavik J, Holzer I, Holub M (2012). Decreased nervonic acid levels in erythrocyte membranes predict psychosis in help-seeking ultra-high-risk individuals. Mol Psychiatry.

[7] Taylor D, Francis T, Guo Y, Brost J, Katavic V, Mietkiewska E (2009). Molecular cloning and characterization of a *KCS* gene from *Cardamine graeca* and its heterologous expression in *Bracssica* oilseeds to engineer high nervonic acid oils for potential medical and industrial use. Plant Biotechnol J.

[8] Guo Y, Mietkiewska E, Francis T, Katavic V, Brost J, Giblin M (2009). Increase in nervonic acid content in transformed yeast and transgenic plants by introduction of a *Lunaria annua* L. 3-ketoacyl- CoA synthase (*KCS*) gene. Plant Mol Biol.

[9] Ye N, Wang X, Li J, Bi C (2017). Assembly and comparative analysis of complete mitochondrial genome sequence of an economic plant *Salix suchowensis*. Peer J.

[10] Birky C (1995). Uniparental inheritance of mitochondrial and chloroplast genes: mechanisms and evolution. Proc Nati Acad Sci.

[11] Cusimano N, Wicke S (2016). Massive intracellular gene transfer during plastid genome reduction in nongreen Orobanchaceae. New Phytol.

[12] Bock R (2017). Witnessing genome evolution: experimental reconstruction of endosymbiotic and horizontal gene transfer. Annu Rev Genet.

[13] Zhao N, Grover C, Chen Z, Wendel J, Hua J (2019). Intergenomic gene transfer in diploid and allopolyploid Gossypium. BMC Plant Biol.

[14] Bi C, Lu N, Xu Y, He C (2020). Characterization and analysis of the mitochondrial genome of common bean (*Phaseolus vulgaris*) by comparative genomic approaches. Int J Mol Sci.

[15] Hsu C, Mullin B (1989). Physical characterization of mitochondrial DNA from cotton. Plant Mol Biol.

[16] Greiner S, Bock R (2013). Tuning a ménage à trois: co-evolution and co-adaptation of nuclear and organellar genomes in plants. Drug Alcohol Depend.

[17] Richardson A, Rice D, Young G, Alverson A, Palmer J (2013). The, “Fossilized” mitochondrial genome of Liriodendron tulipifera: ancestral gene content and order, ancestral editing sites, and extraordinarily low mutation rate. BMC Biol.

[18] Skippington E, Barkman T, Rice D, Palmer J (2015). Miniaturized mitogenome of the parasitic plant Viscum scurruloideum is extremely divergent and dynamic and has lost all *nad* genes. Proc Natl Acad Sci U S A.

[19] Sloan D, Alverson A, Chuckalovcak J, Wu M, Mccauley D (2012). Rapid evolution of enormous, multichromosomal genomes in flowering plant mitochondria with exceptionally high mutation rates. PLoS Biol.

[20] Guo W, Felix G, Fan W, Young G (2016). *Ginkgo* and *Welwitschia* Mitogenomes reveal extreme contrasts in gymnosperm mitochondrial evolution. Mol Biol Evol.

[21] Bergthorsson U, Adams K, Thomason B, Palmer J (2003). Widespread horizontal transfer of mitochondrial genes in flowering plants. Nature..

[22] Wynn E, Christensen A (2019). Repeats of unusual size in plant mitochondrial genomes: identification, incidence and evolution. G3: genes, genomes. Genetics..

[23] Ma Q, Li S, Bi C, Hao Z, Sun C, Ye N (2017). Complete chloroplast genome sequence of a major economic species, *Ziziphus jujuba* (Rhamnaceae). Curr Genet.

[24] Seok J, Kim A, Wang J, Hee K, Iksoo L (2019). Single-nucleotide polymorphism markers in mitochondrial genomes for identifying Varroa destructor-resistant and -susceptible strains of Apis mellifera (Hymenoptera: Apidae). Mitochondrial DNA Part A, DNA Mapp Seq Anal.

[25] Mwamuye M, Obara I, Khawla E, Odongo D, Bakheit M, Jongejan F (2020). Unique mitochondrial single nucleotide polymorphisms demonstrate resolution potential to discriminate *Theileria parva* vaccine and Buffalo-derived strains. Life..

[26] Bi C, Paterson A, Wang X, Xu Y, Wu D, Qu Y (2016). Analysis of the complete mitochondrial genome sequence of the diploid cotton *Gossypium raimondii* by comparative genomics approaches. Biomed Res Int.

[27] Choi I, Schwarz E, Ruhlman T, Khiyami M, Sabir J, Hajarah N (2019). Fluctuations in Fabaceae mitochondrial genome size and content are both ancient and recent. BMC Plant Biol.

[28] Yang J, Wariss H, Tao L, Zhang R, Yun Q, Hollingsworth P (2019). De *novo* genome assembly of the endangered *Acer yangbiense*, a plant species with extremely small populations endemic to Yunnan Province, China. Gigascience.

[29] Ma Q, Wang Y, Zhu L, Bi C, Li S, Li S (2019). Characterization of the complete chloroplast genome of *Acer truncatum* bunge (Sapindales: Aceraceae): a new woody oil tree species producing nervonic acid. Biomed Res Int.

[30] Kozik A, Rowan B, Lavelle D, Berke L, Schranz M, Michelmore R (2019). The alternative reality of plant mitochondrial DNA: one ring does not rule them all. PLoS Genet.

[31] Cheng Y, He X, Priyadarshani S, Wang Y, Ye L, Qin Y (2021). Assembly and comparative analysis of the complete mitochondrial genome of *Suaeda glauca*. BMC Genomics.

[32] Chang S, Yang T, Du T, Huang Y, Chen J (2021). Mitochondrial genome sequencing helps show the evolutionary mechanism of mitochondrial genome formation in *Brassica*. BMC Genomics.

[33] Sloan D, Wu Z, Sharbrough J (2018). Correction of persistent errors in *Arabidopsis* reference mitochondrial genomes. Plant Cell.

[34] Kubo T, Nishizawa S, Sugawara A, Itchoda N, Estiati A, Mikami T (2000). The complete nucleotide sequence of the mitochondrial genome of sugar beet (*Beta vulgaris* L.) reveals a novel gene for tRNACys (GCA). Nucleic Acids Res.

[35] Zhang C, Ma H, Sanchez-Puerta MV, Li L, Xiao J (2020). Horizontal gene transfer has impacted *cox1* gene evolution in Cassytha filiformis. J Mol Evol.

[36] Xiong Y, Lei X, Bai S, Xiong Y, Liu W, Wu W (2021). Genomic survey sequencing, development and characterization of single- and multi-locus genomic SSR markers of *Elymus sibiricus* L. BMC Plant Biol.

[37] Liu L, Fan X, Tan P, Wu J, Zhang H, Han C (2021). The development of SSR markers based on RNA-sequencing and its validation between and within *Carex* L. species. BMC Plant Biol.

[38] Gualberto J, Mileshina D, Wallet C, Niazi A, Weber-Lotfi F, Dietrich A (2014). The plant mitochondrial genome: dynamics and maintenance. Biochimie..

[39] Guo W, Zhu A, Fan W, Mower J (2017). Complete mitochondrial genomes from the ferns *Ophioglossum californicum* and *Psilotum nudum* are highly repetitive with the largest organellar introns. New Phytol.

[40] Alverson AJ (2010). Insights into the evolution of mitochondrial genome size from complete sequences of *Citrullus lanatus* and *Cucurbita pepo* (Cucurbitaceae). Mol Biol Evol.

[41] Chang S, Wang Y, Lu J, Gai J, Li J, Chu P (2031). The mitochondrial genome of soybean reveals complex genome structures and gene evolution at intercellular and phylogenetic levels. PLoS One.

[42] Notsu Y, Masood S, Nishikawa T, Kubo N, Akiduki G, Nakazono M (2002). The complete sequence of the rice (*Oryza sativa* L.) mitochondrial genome: frequent DNA sequence acquisition and loss during the evolution of flowering plants. Mol gen. Genomics..

[43] Mower J (2005). PREP-Mt: predictive RNA editor for plant mitochondrial genes. BMC Bioinformatics.

[44] Timmis J, Ayliffe M, Huang C, Martin W (2004). Endosymbiotic gene transfer: organelle genomes forge eukaryotic chromosomes. Nat Rev Genet.

[45] Nguyen V, Giang V, Waminal N, Park H, Kim N, Jang W (2000). Comprehensive comparative analysis of chloroplast genomes from seven *Panax* species and development of an authentication system based on species-unique single nucleotide polymorphism markers. J Ginseng Res.

[46] Martin W, Stoebe B, Goremykin V, Hansmann S, Hasegawa M, Kowallik KV (1998). Gene transfer to the nucleus and the evolution of chloroplasts. Nature..

[47] Zhao N, Wang Y, Hua J (2018). The roles of mitochondrion in intergenomic gene transfer in plants: a source and a pool. Int J Mol Sci.

[48] Rice D, Alverson A, Richardson A, Young G, Sanchez-Puerta M, Munzinger J (2013). Horizontal transfer of entire genomes via mitochondrial fusion in the angiosperm *Amborella*. Science..

[49] Smith D, Crosby K, Lee R (2011). Correlation between nuclear plastid DNA abundance and plastid number supports the limited transfer window hypothesis. Genome Biol Evol.

[50] Sebbenn A, Blanc-Jolivet C, Mader M, Meyer-Sand B, Degen B (2019). Nuclear and plastidial snp and indel markers for genetic tracking studies of *jacaranda copaia*. Conservation Genet Resour.

[51] Xu T (1998). The systematic evolution and distribution of the genus *Acer*.

[52] Chase F, Freudenstein J (2001). Chase analysis of mitochondrial *nad1*b-c intron sequences in Orchidaceae: utility and coding of length-change characters. Syst Bot.

[53] Zhang T, Wang Z, Xu L, Zhou K (2005). Application of mitochondrial *nad 1* intron 2 sequences to molecular identification of some species of *dendrobium* Sw. Chinese Traditional and Herbal Drugs.

[54] Pan Z, Ren X, Zhao H, Liu L, Tan Z, Qiu F (2019). A mitochondrial transcription termination factor, zmsmk3, is required for nad1 intron4 and nad4 intron1 splicing and kernel development in maize. G3: Genes Genomes. Genetics.

[55] Koren S, Walenz B, Berlin K, Miller J, Phillippy A (2017). Canu: scalable and accurate long-read assembly via adaptive k-mer weighting and repeat separation. Genome Res.

[56] Li H (2018). Minimap2: pairwise alignment for nucleotide sequences. Bioinformatics.

[57] Walker B, Abeel T, Shea T, Priest M, Abouelliel A, Sakthikumar S (2014). Pilon: an integrated tool for comprehensive microbial variant detection and genome assembly improvement. PLoS One.

[58] Lowe T, Eddy S (1997). tRNAscan-SE: a programfor improved detection of transfer RNA genes in genomic sequence. Nucleic Acids Res.

[59] Kumar S, Stecher G, Li M, Knyaz C, Tamura K (2018). MEGA X: molecular evolutionary genetics analysis across computing platforms. Mol Biol Evol.

[60] Greiner S, Lehwark P, Bock R (2019). OrganellarGenomeDRAW (OGDRAW) version 1.3.1: expanded toolkit for the graphical visualization of organellar genomes. Nucleic Acids Res.

[61] Kurtz S, Choudhuri J, Enno O, Chris S, Jens S, Robert G (2001). REPuter: the manifold applications of repeat analysis on a genomic scale. Nucleic Acids Res.

[62] Thiel T, Michalek W, Varshney R, Graner A (2003). Exploiting EST databases for the development and characterization of gene-derived SSR-markers in barley (*Hordeum vulgare* L.). Theor Appl Genet.

[63] Librado RJ (2009). DnaSP v5: a software for comprehensive analysis of DNA polymorphism data. Bioinformatics..

[64] Edgar R (2004). MUSCLE: multiple sequence alignment with high accuracy and high throughput. Nucleic Acids Res.

