# Supplementary material for: Assembly and comparative analysis of the first complete mitochondrial genome of Acer truncatum Bunge: a woody oil-tree species producing nervonic acid
Source: BMC Plant Biol. 2022 Jan 13;22:29. doi: 10.1186/s12870-021-03416-5 (PMC8756732; doi:10.1186/s12870-021-03416-5)
Supplement: Supplementary file 1 — Additional file 1: Figure S1. Reversible reorganization of the A. truncatum mitgenome may produce subgenomic circles by large repeats. The same colour triangles represent the pairs of large repeats. [file 12870_2021_3416_MOESM1_ESM.doc]

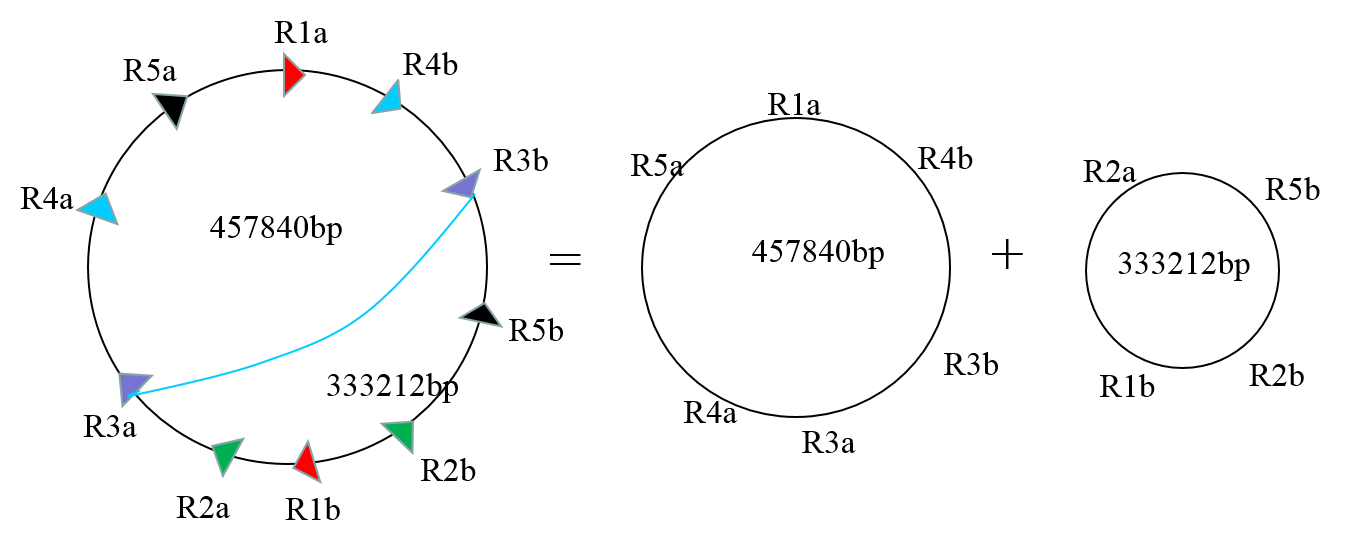


**Fig.S1. Reversible reorganization of the *A. truncatum* mitgenome may produce subgenomic circles by large repeats. The same colour triangles represent the pairs of large repeats.**
