# Supplementary material for: Assembly and comparative analysis of the first complete mitochondrial genome of Acer truncatum Bunge: a woody oil-tree species producing nervonic acid
Source: BMC Plant Biol. 2022 Jan 13;22:29. doi: 10.1186/s12870-021-03416-5 (PMC8756732; doi:10.1186/s12870-021-03416-5)
Supplement: Supplementary file 2 — Additional file 2: Figure S2. The type of detected repeats and the frequency distribution of lengths in the A. yangbiense. [file 12870_2021_3416_MOESM2_ESM.doc]

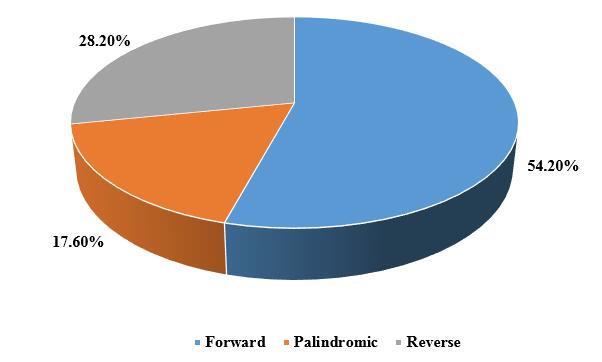


A


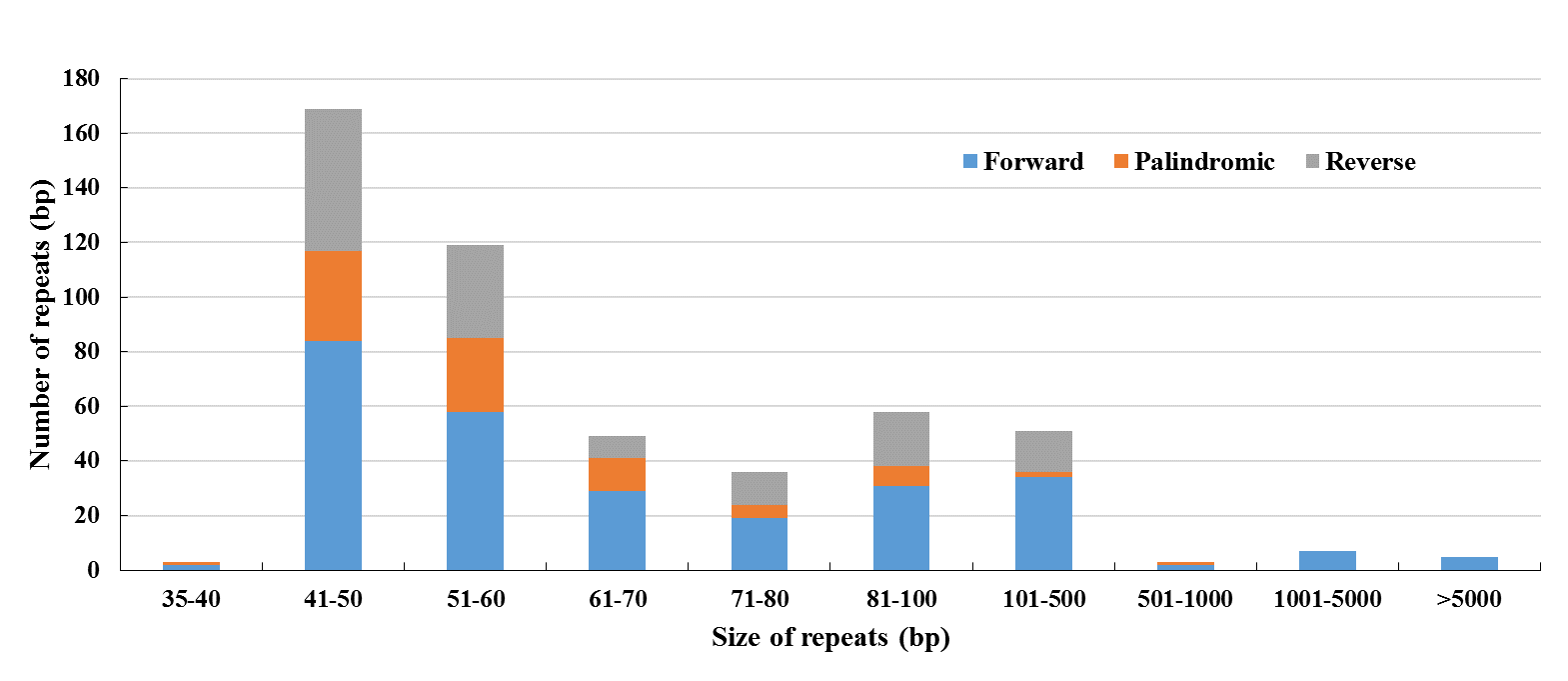


B

**Fig. S2 A.** The type and proportion of detected repeats in the *A. yangbiense* mitogenome; B. The frequency distribution of repeat lengths in the *A. yangbiense* mitogenome.
