# Supplementary figures and images for: Assembly and comparative analysis of the first complete mitochondrial genome of Acer truncatum Bunge: a woody oil-tree species producing nervonic acid
Source: BMC Plant Biol. 2022 Jan 13;22:29. doi: 10.1186/s12870-021-03416-5 (PMC8756732; doi:10.1186/s12870-021-03416-5)

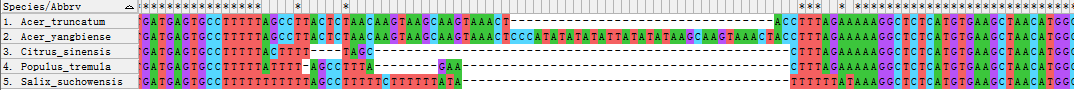


Fig. S3 Alignment of the *NAD1* intron sequence with MEGA-X.

Supplement: Supplementary file 3 — Additional file 3: Figure S3. Alignment of the NAD1 intron sequence with MEGA-X. [file 12870_2021_3416_MOESM3_ESM.doc]

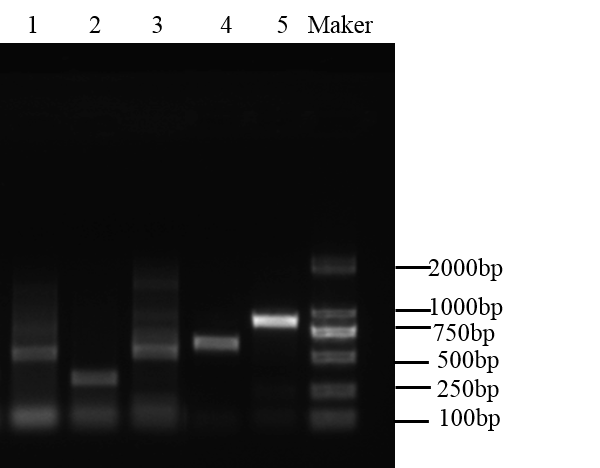


Fig. S4 Agarose gel electrophoresis of PCR product for contig connecting verification

Supplement: Supplementary file 4 — Additional file 4: Figure S4. Agarose gel electrophoresis of PCR product for contig connecting verification [file 12870_2021_3416_MOESM4_ESM.doc]
