# Supplementary material for: Assembly and comparative analysis of the first complete mitochondrial genome of Acer truncatum Bunge: a woody oil-tree species producing nervonic acid
Source: BMC Plant Biol. 2022 Jan 13;22:29. doi: 10.1186/s12870-021-03416-5 (PMC8756732; doi:10.1186/s12870-021-03416-5)
Supplement: Supplementary file 6 — Additional file 6: Table S2. The large repeats (> 1 kb) by rearrangements could produce two subgenomic circles in A. truncatum mitogenome. [file 12870_2021_3416_MOESM6_ESM.doc]

**Table S2. The direct or reverse repeats (>1 kb) that form subgenomic circles in the *A. truncatum* mitogenome.**

| Name | Start1 | End1 | Name | Start2 | End2 | Length | Similarity (%) |
| --- | --- | --- | --- | --- | --- | --- | --- |
| R1a | 720609 | 749055 | R1b | 585001 | 556573 | 28452 | 99.902 |
| R2a | 424259 | 431032 | R2b | 318164 | 311391 | 6774 | 100 |
| R3a | 479068 | 482574 | R3b | 21228 | 24734 | 3507 | 100 |
| R4a | 615777 | 619031 | R4b | 1 | 3256 | 3256 | 99.969 |
| R5a | 686260 | 688127 | R5b | 42384 | 40515 | 1870 | 99.626 |
