# Supplementary material for: Assembly and comparative analysis of the first complete mitochondrial genome of Acer truncatum Bunge: a woody oil-tree species producing nervonic acid
Source: BMC Plant Biol. 2022 Jan 13;22:29. doi: 10.1186/s12870-021-03416-5 (PMC8756732; doi:10.1186/s12870-021-03416-5)
Supplement: Supplementary file 8 — Additional file 8: Table S4. Details regarding the mitochondira genome sequences used for the phylogenetic analysis. [file 12870_2021_3416_MOESM8_ESM.doc]

**Table S4**. Details regarding the mitochondira genome sequences used for the phylogenetic analysis.

| No. | Taxon | | Family | | Order | | GenBank Accession number |
| --- | --- | --- | --- | --- | --- | --- | --- |
| 1 | *Acacia ligulata* | | *Caesalpinioideae* | | Fabales | | NC_040998.1 |
| 2 | *Leucaena trichandra* | | *Caesalpinioideae* | | Fabales | | NC_039738.1 |
| 3 | *Senna tora* | *Caesalpinioideae* | | Fabales | | NC_038053.1 | |
| 4 | *Haematoxylum brasiletto* | *Caesalpinioideae* | | Fabales | | MN017229.1 | |
| 5  6  7  8  9  10  11  12  13  14  15  16  17  18  19  20  21  22  23  24  25  26 | *Libidibia coriaria*  T*amarindus indica*  *Ammopiptanthus mongolicus*  *Medicago truncatula*  *Glycine max*  *Vigna radiata*  *Cercis canadensis*  *Acer truncatum*  *Acer yangbiense*  *Citrus sinensis*  *Populus tremula*  *Salix suchowensis*  *Gossypium raimondii*  *Carica papaya*  *Arabidopsis thaliana*  *Brassica napus*  *Raphanus sativus*  *Vitis vinifera*  *Triticum aestivum*  *Sorghum bicolor*  *Zea mays*  *Ginkgo biloba* | *Caesalpinioideae*  *Detarioideae*  *Papilionoideae*  *Papilionoideae*  *Papilionoideae*  *Papilionoideae*  *Cercidoideae*  *Acereae*  *Acereae*  *Rutaceae*  *Salicaceae*  *Salicaceae*  *Malvoideae*  *Caricaceae*  *Brassicaceae*  *Brassicaceae*  *Brassicaceae*  *Vitaceae*  *Poaceae*  *Poaceae*  *Poaceae*  *Ginkgoaceae* | | Fabales  Fabales  Fabales  Fabales  Fabales  Fabales  Fabales  Sapindales  Sapindales  Sapindales  Malpighiales  Malpighiales  Malvales  Brassicales  Brassicales  Brassicales  Brassicales  Vitales  Poales  Poales  Poales  Ginkgoales | | MN017228.1  MN017227.1  MG011535.1  NC_029641.1  NC_020455.1  NC_015121.1  MN017226.1  MZ318049  CM017774.1  NC_037463.1  KT337313.1  NC_029317.1  NC_029998.1  NC_012116.1  NC_037304.1  NC_008285.1  NC_018551.1  NC_012119.1  NC_036024.1  NC_008360.1  NC_007982.1  NC_027976.1 | |
