# Supplementary material for: Assembly and comparative analysis of the first complete mitochondrial genome of Acer truncatum Bunge: a woody oil-tree species producing nervonic acid
Source: BMC Plant Biol. 2022 Jan 13;22:29. doi: 10.1186/s12870-021-03416-5 (PMC8756732; doi:10.1186/s12870-021-03416-5)
Supplement: Supplementary file 9 — Additional file 9: Table S5. Details regarding the primers used to develop the NAD1 intron marker. [file 12870_2021_3416_MOESM9_ESM.doc]

**Table S5. Details regarding the primers used to develop the *NAD1* intron marker.**

| **Primer name** | **Primer sequence (5’>3’)** |
| --- | --- |
| Nad1-F1 | AACGTGAGAGCAAGGGATCA |
| Nad1-R1 | AGGACATTTGGTGAGGAGCA |
