# Supplementary material for: Assembly and comparative analysis of the first complete mitochondrial genome of Acer truncatum Bunge: a woody oil-tree species producing nervonic acid
Source: BMC Plant Biol. 2022 Jan 13;22:29. doi: 10.1186/s12870-021-03416-5 (PMC8756732; doi:10.1186/s12870-021-03416-5)
Supplement: Supplementary file 10 — Additional file 10: Table S6. Primers for contig connecting verification [file 12870_2021_3416_MOESM10_ESM.doc]

**Table S6. Primers for contig connecting verification**

| Name | Primer sequences (5’>3’) | TM(℃) | Product length | Contig connections |
| --- | --- | --- | --- | --- |
| 1 | ATCAATCCCGTTGCCACATC | 55 | 486 | Contig 1-2 |
|  | ACGACCATCAGAGTCCTCCC |  |  |  |
| 2 | TACCTAACTCTAACGGAAACG | 53 | 354 | Contig 2-3 |
|  | AGACGGCGAGAACTTACGAC |  |  |  |
| 3 | ACCTAACTCTAACGGAAACG | 53 | 516 | Contig 3-4 |
|  | GTCAGTAAATCCCTCACAGC |  |  |  |
| 4 | TTCATCAAGCCATTTCTCGC | 54 | 629 | Contig 4-5 |
|  | CTGCCGTCCCATTCTTGAGT |  |  |  |
| 5 | GATTGCTCCCGCTTCCTCAC | 56 | 894 | Contig 1-5 |
|  | AGCCATCTTATCCACTGAACT |  |  |  |
